# In need of robust evidence of non-association of pregestational and early pregnancy SARS-CoV-2 infections with congenital anomalies

**DOI:** 10.1016/j.eclinm.2024.102729

**Published:** 2024-07-13

**Authors:** Athina Samara, Vivienne Souter, Conrado Milani Coutinho, Asma Khalil

**Affiliations:** a Department of Women's and Children's Health, Karolinska Institutet, Stockholm, Sweden; b Astrid Lindgren Children's Hospital, Karolinska University Hospital, Stockholm, Sweden; c FUTURE, Center for Functional Tissue Reconstruction, University of Oslo, Oslo, Norway; d Foundation for Health Care Quality, Seattle, WA, USA; e Hospital das Clínicas, Ribeirão Preto Medical School, University of São Paulo, Ribeirão Preto, Brazil; f Fetal Medicine Unit, St George's University Hospitals NHS Foundation Trust, University of London, London, UK; g Vascular Biology Research Centre, Molecular and Clinical Sciences Research Institute, St George's University of London, London, UK; h Royal College of Obstetricians and Gynaecologists, London, UK

**Keywords:** Congenital anomalies, Pregnancy, Maternal infection, Neural tube defect, NTDs, COVID-19, SARS-CoV-2

## Abstract

SARS-CoV-2 infection during pregestational and early pregnancy periods has an unclear impact on fetal development. Although vertical transmission is rare, potential effects on the developing fetal brain are plausible. However, robust evidence linking maternal SARS-CoV-2 infection to congenital anomalies is limited due to inadequate tracking of infection history and methodological flaws in published studies. This is further complicated by limitations, such as restricted testing access and undiagnosed infections, particularly in low- and middle-income countries. Most data focus on hospitalized women near term, lacking information on first- and second-trimester infections. Thus, an accurate assessment of the impact of COVID-19 on congenital anomalies is essential. It should however be emphasised that we have robust evidence that vaccination against COVID-19 before or during early pregnancy is not associated with malformations, ruling out any role of COVID-19 vaccines in these increased rates of congenital abnormalities. This viewpoint discusses findings from surveillance registries, highlights study limitations, and offers research recommendations to inform clinical guidelines and public health strategies, aiming to mitigate the effects of viral infections on early neurodevelopment.

## Introduction

The impact of pregestational and maternal SARS-CoV-2 infection during early pregnancy on the developing fetus is poorly understood. Although vertical transmission is rare, an effect on the developing fetal brain remains plausible. Robust evidence on any association between maternal SARS-CoV-2 infection and the risk of congenital anomalies in offspring is, however, limited. This is due to inadequate tracking of pregestational maternal SARS-CoV-2 infection history and infection during early pregnancy, along with methodological limitations in relevant published studies.

The significant proportion of undetected or undiagnosed SARS-CoV-2 infections during early pregnancy, particularly in low- and middle-income countries (LMICs), due to limited access to testing and information, further complicates this issue.^1^ Most available data pertain to hospitalised pregnant women near term, with very little information on the outcomes of first- and second-trimester infections separately.^2^ Therefore, there is a clear need to accurately assess the impact of COVID-19 on congenital anomalies.

## Impact on maternal infection

Pregnancies affected by COVID-19 were mostly mild, asymptomatic, and sometimes undetected. The lower access to health services and poor case registration during the pandemic hinder researchers' ability to link maternal health records at infection to newborns at birth. However, fever, that was frequently reported in both pregnant and non-pregnant individuals with COVID-19, poses risks during pregnancy.^3^ It can potentially cause vascular disruptions, placental infarction, and fetal developmental anomalies like neural tube defects (NTDs), congenital heart defects, oral clefts, growth restriction, and fetal loss.^4^, ^5^, ^6^ Notably, early pregnancy infections are crucial as the embryo is particularly vulnerable to malformations during neural tube formation, occurring from day 20 to 28 of human development.^7^

The cell tropism and persistence of SARS-CoV-2 has now been documented^8^ and viral presence in ovarian stromal and endometrial gland epithelial and stromal cells in post-mortem samples of unvaccinated women has been demonstrated. These findings could infer the potential for previous SARS-CoV-2 infection to have a role in complications in future pregnancies. COVID-19 has been associated with an increased risk of adverse pregnancy outcomes and *in vitro* studies have shown that SARS-CoV-2 can infect granulosa and cumulus cells in the ovaries.^9^ However, we have scant data regarding viral persistence in germ cells and reproductive tissue in pregnant women post-COVID-19. Although the high percentage of global vaccination is reassuring, this remains an understudied, possibly strain-dependent matter.

## Surveillance registries

**Fig. 1:**
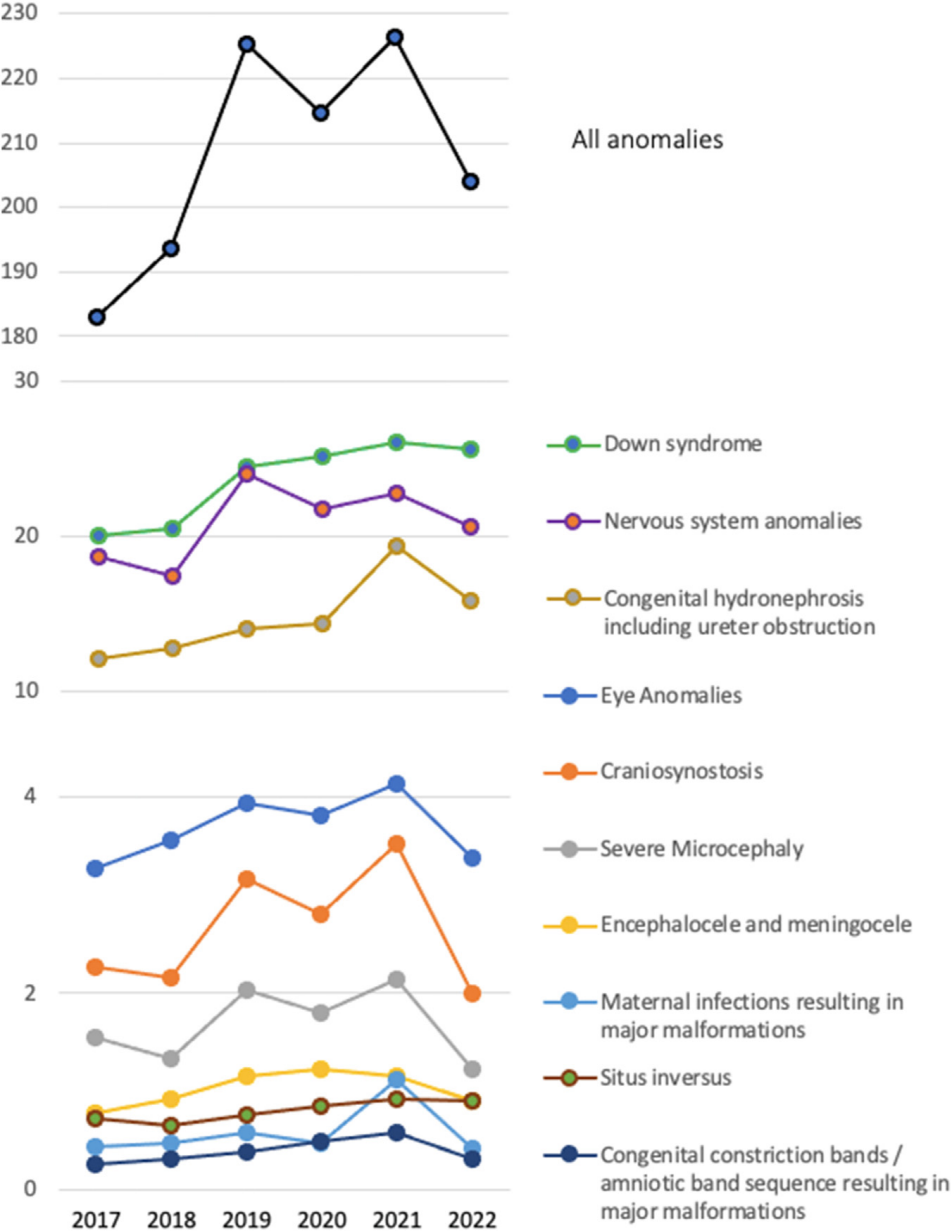
Prevalence of selected congenital anomalies per 10,000 births using data from all registries of EUROCAT from 2017 to 2022. Lines indicate prevalence from data for all cases, i.e., live births and stillbirths from 20 weeks-gestation, and cases corresponding to termination of pregnancy due to congenital anomaly.

Data from the European Surveillance of congenital anomalies (EUROCAT) platform exclusive of genetic anomalies covering 2017–2022, reveal a rise in selected abnormalities, both in the prevalence per 10,000 births of all congenital anomalies in 2021 compared to previous years, and since 2017 (Fig. 1).^10^ Notably, the trend is reversed in 2022. While the prevalence of Down syndrome diagnoses per 10,000 births has increased since 2020 (from 24.44 in 2019, to 25.2 in 2020 and 26.8 in 2021), so have the pregnancy terminations registered at EUROCAT (from 14.59 in 2019, to 14.98 in 2020 and 16.84 in 2021), indicating that the increased prevalence cannot be attributed solely to limited access to maternal health facilities for pregnant women (Fig. 1).

Furthermore, the prevalence of congenital hydronephrosis including ureter obstruction, eye anomalies, craniosynostosis, and maternal infections resulting in major malformations was increased in 2021. The prevalence of nervous system anomalies, encephalocele and meningocele, and severe microcephaly were similar or lower in 2019. There was a slight increase in the prevalence of situs inversus from 2019 to 2021 (0.72, 0.85, 0.92 in 2019, 2020 and 2021, respectively) and of congenital restriction bands (Fig. 1).

**Fig. 2:**
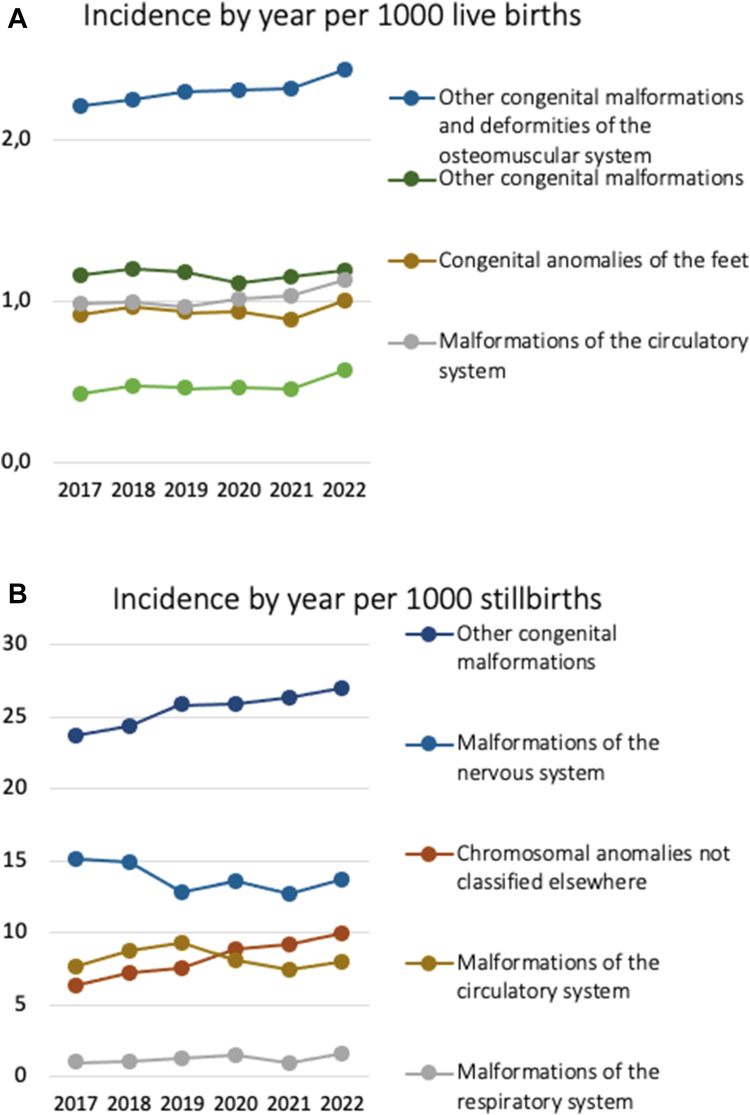
Data from Brazil from 2017 to 2022 were used to calculate the incidence of congenital anomalies per 1000 live births (**A**) and 1000 stillbirths (**B**).

Published reports reveal a concerning trend of congenital anomalies in countries where abortions are illegal or inaccessible to pregnant women (Table 1). This complicates the assessment of whether COVID-19 is solely responsible for the observed increase in congenital anomalies, as limited access to termination services hinders thorough investigations into potential causative factors. In Brazil, under the current penal code, abortion is criminalized except in specific circumstances. Considering this, and given the public availability of congenital anomalies data among live births and stillbirths in Brazil, we examined data from 2017 to 2022 to calculate the annual incidence of selected congenital anomalies among live births (Fig. 2A) (http://tabnet.datasus.gov.br/cgi/tabcgi.exe?sinasc/cnv/nvuf.def) and stillbirths (Fig. 2B) (https://svs.aids.gov.br/daent/centrais-de-conteudos/paineis-de-monitoramento/mortalidade/infantil-e-fetal/), by body system. Of note, during the pandemic years, increased trends in the number of malformations of the circulatory, digestive and osteomuscular systems, as well as chromosomal anomalies were noted among livebirths. The same trend was noted concerning the total number of malformations and chromosomal anomalies among stillbirths.

**Table 1:** Studies and reports on congenital anomalies related to SARS-CoV-2 maternal infections.

Location/Country	Period	Sample	Findings	Maternal infection severity	Source
Germany	February 2020–April 2023	8032 pregnancies	1.96% prevalence of congenital malformations	Available	11
Scotland	May 2020–April 2022	1574 babies from pregnancies affected by COVID-19 vs 4722 healthy (control) babies	No association between SARS-CoV-2 infection and congenital anomalies	No maternal infection, no miscarriage data	12
Quebec, Canada	Prepandemic (January 1, 2017–March 12, 2020) vs pandemic (March 13, 2020–March 31, 2022)	420,222 neonates	Congenital anomalies in 29,263 newborns (7%). Births with the first trimester in the pandemic had higher rates of microcephaly diagnosis	No maternal infection, no miscarriage data	13
Iran	Pandemic (November 1, 2020–February 28, 2021) compared to pre-pandemic period (November 1, 2019–February 29, 2020)	Iranian Maternal and Neonatal Network data; 347,839 ‘pandemic vs 395,728 pre-pandemic newborns	Significant increase in the incidence of congenital anomalies (2012 vs 1868) (P < 0.00001)	No maternal infection, no miscarriage data	14
Iraq	2014–2021	Health directorate statistics from January 2013 to December 2021. And 18 patient case-series	Upward trend in NTDs from 27 per 10,000 in 2014 to 53 per 10,000 births in 2021, with incidence increase in 2020	Fever in 1st trimester, 83.3%. Mild COVID-19 77.8%, moderate severity 22.2%. No miscarriage data	15
Zakho Maternity hospital, Iraq	January 1, 2020–January 1, 2022	68 cases of NTDs (single-site case series)	68 NTD cases in newborns, of which 29 were associated with maternal infection in the first trimester (30 in 2nd, 9 in 3rd)	Mild 75%; Moderate 22.1%, Severe 2.9%; Anaemia and fever in the 1st trimester: 79.4%. No miscarriage data	16
Abha Hospital, Saudi Arabia Kingdom	2018–2022, with descriptive cross-sectional record-based analysis of September–December 2023	14,664 births, (14,647 live births and 17 stillbirths)	Five-year prevalence of congenital anomalies was 3.21% (472, 12 of which were stillbirths, 6 died in first month). Prevalence increased to 4.02% (127 out of 3158 births) in 2022	No maternal infection, no miscarriage data	17
Shanghai (IPMCH) and Changsha. (HPM) China	January 1, 2014–December 31, 2022 & January 1–July 31, 2023	IPMCH site; 151, 368 (2014–2022): 8321 (2023) HPM site; 287,034 (2014–2022): 15,425 (2023)	4.2-fold increase in situs inversus cases during the first 7 months of 2023 compared to 2014–2022 average annual incidence (23.6 vs 5.6)	No maternal infection, no miscarriage data	18
Jinan, China	January–May 2022 & January–May 2023.	3956 COVID-19 patients, 4400 uninfected	25 mothers with situs inversus fetuses, 22 had COVID-19 and 3 without recent infection. The analysis showed a strong link between COVID-19 and a higher fetus situs inversus (P < 0.001, odds ratio 8.196)	Fever duration 2.06 ± 1.52 days; highest body temperature 38.94 ± 0.51 °C, gestational infection week 6.13 ± 2.4 weeks	19
Maharashtra, India	January 1, 2020–December 31, 2021	516,655 babies examined in 2021	101 (0.02%) NTDs in 2021; four-fold statistically significant increase as per records of national health programme Rashtriya Bal Swasthya Karyakram (P < 0.001)	No maternal infection, no miscarriage data	21

IPMCH, International Peace Maternity and Child Health Hospital of China Welfare Institute; HPM, Hunan Provincial Maternal and Child Health Care Hospital; NTDs, neural tube defects.

## Knowledge gaps due to inconsistent methodologies

The limitations of current studies investigating the link between SARS-CoV-2 infection and congenital anomalies, however, are not restricted to incomplete reporting and registry data, such as in the case of European countries that often delay reporting to EUROCAT, leading to incomplete registries.

Methodological differences have also been noted and summarised in Table 1, among studies were identified through searches of PubMed with the search terms “congenital anomalies”, “COVID-19”, “SARS-CoV-2”, “neural tube defect”, “NTDs”, and “pregnan” from 2020 until June 2024. Relevant articles were also identified through searches of the authors’ files. The final reference list was generated based on relevance to the broad scope of this viewpoint.

The German CRONOS study found a 1.96% prevalence of congenital malformations among 8032 pregnancies but did not link COVID-19 to these anomalies.^11^ A Scottish national cohort study found no association between maternal SARS-CoV-2 infection and congenital anomalies, regardless of their birth status, and irrespective of pregnancy outcomes, in 1574 affected pregnancies compared to 4722 controls.^12^ However, it did not assess microcephaly, infection severity, or fever, and potential misclassification due to testing restrictions.

Furthermore, sample urgency assessment due to the pandemic period should be taken into consideration. A Canadian study of 420,222 neonates compared pre-pandemic and pandemic births, finding no increase in congenital anomalies from first-trimester SARS-CoV-2 infections.^13^ However, microcephaly rates were higher during late-pandemic births, possibly due to increased detection.^13^ The challenge of tackling incomplete records of the history of pregestational maternal SARS-CoV-2 infection and infection during early pregnancy, has led other researchers to approach the estimation of congenital anomalies using different methods. This added to the variability in the population cohorts assessed, study designs, and investigation context. An Iranian study, for example, reported a significant increase in congenital anomalies during the pandemic,^14^ while studies from Iraq showed an upward trend in NTDs.^15^^,^^16^ Furthermore, similar to others, the Iranian study has no data on the history of maternal COVID-19 during pregnancy, as it had not been registered. Similarly, in India, as there is no national registry for birth defects, the estimation of the prevalence of birth defects is difficult.

Studies from Saudi Arabia^17^ and China highlighted increases in specific congenital conditions, such as NTDs and situs inversus.^18^^,^^19^ This further illustrates the geographical and methodological variances, as the findings did not align with the incidence of fetal situs inversus reported during the COVID-19 pandemic in the Scandinavian countries of Sweden, Denmark, and Norway.^20^ Maternal health and nutritional status should also be factored in, as depending on the location there could be potential confounding effects of maternal malnutrition, particularly folic acid deficiency. This was observed both in the Saudi Arabian study, and a study from India reporting a significant increase in NTD during the pandemic.^21^ As also exemplified by some of these studies, rural and urban areas could have been affected uniformly by the pandemic. The impact in these regions was different, during the pandemic period, where there was a greater difficulty in accessing in-person medical care and imaging exams, especially in LMIC, where many of these studies were carried out.

Finally, the heightened surveillance and detection during the pandemic may have contributed to the increased detection and reporting of certain anomalies. These limitations stress the need for more comprehensive and methodologically robust studies, beyond self-reporting, to accurately assess the impact of COVID-19 on congenital anomalies. They further accentuate the need to consider factors like maternal health and access to health services including termination services, socioeconomic background, and nutritional status, facilitated by thorough reporting and registry systems.

## Future directions & recommendations

Epidemiological studies indicate that prenatal exposure to certain viruses may affect early neurodevelopment. Thus, it is imperative to investigate whether prenatal SARS-CoV-2 infection resulting in placental infection and/or vertical transmission leads to more severe neurodevelopmental outcomes in offspring. In a US-based observational study from June 2020 to November 2021, infants were compared to pre-pandemic controls (studied from March 2014 to February 2020).^22^ Among 210 infants (55 from COVID-19 unexposed mothers, 47 from COVID-19-positive mothers, and 108 pre-pandemic healthy controls), those born to COVID-19-positive mothers showed increased cortical grey matter volume and accelerated sulcal depth in the frontal lobe compared to controls. Additional differences in infants of COVID-19 unexposed mothers suggest that both viral and non-viral stressors associated with the pandemic may impact early development, warranting ongoing follow-up.^23^

Although SARS-CoV-2 is unlikely to be teratogenic, the long-term risk of neurodevelopmental issues in children exposed to the virus *in utero* remains unclear. In some women, SARS-CoV-2 infection may induce the release of proinflammatory cytokines in the placenta, potentially impacting fetal development, including neurodevelopment.^24^^,^^25^

Future studies should address several crucial questions regarding the impact of various factors on neurodevelopmental outcomes in offspring. These questions include how variant strain and severity of maternal illness influence the risk of neurodevelopmental abnormalities in children, whether fetal sex plays a role in neurodevelopmental risk, and the effects of other prenatal exposures on the incidence of NTDs. Other first-trimester viral infections, such as Zika virus,^26^, ^27^, ^28^ influenza^29^ and rubella,^30^ are known to be associated with NTDs. Future studies on early SARS-CoV-2 infections should consider these factors, as fever or hypoxia can both increase the vulnerability of the developing central nervous system (CNS) to malformations.

In addition, the cause of miscarriage in the first trimester is often unknown. Studies have revealed that maternal viral infections do not necessarily need to bypass the placental membrane to affect fetal development. Potential maternal immune responses and cytokines induced by intrauterine viral infections could cause adverse outcomes for the fetus. Exploring the potential of cell, tissue or organoid models to understand placental dysfunction and neurodevelopmental risks, and to guide interventions and therapeutics, particularly concerning SARS-CoV-2 infection in early pregnancy, is another essential avenue for research. By uncovering epigenetic and molecular mechanisms post-infection, 3D brain organoid and trophoblast models may help to understand how infections and the induction of apoptosis in specific cell populations affect placental function. Combining them with simulations of hypoxia and fever we could molecularly characterise the complications relevant to nutrient and oxygen transfer from mother to fetus. These models could thus provide us with insights into interactions at the maternal–fetal interface, assess risks of vertical transmission of infections, and evaluate the safety of medications and vaccines on placental cells. They can also provide crucial insights into how viral infections might impact early fetal development.

In addition, neuronal differentiation studies could identify more specific effects, such as eye development,^31^ and aberrant morphogen distribution and cilia dysfunction, that could lead to situs inversus.^32^ Comparing the transcriptional^33^ and epigenetic landscapes of umbilical cord cells from neonates born to uninfected vs mothers infected at different trimesters, with future blood samples, could also enhance our understanding of neurodevelopmental outcomes in children exposed to prenatal SARS-CoV-2 infection. Further bioinformatics analyses could also provide associations to cell types, target genes and pathways relative to miscarriages.^34^ This knowledge is crucial for informing future preventive and therapeutic strategies. Additionally, these findings can inform public health strategies and clinical guidelines to mitigate the effects of viral infections on early neurodevelopment.

## Conclusion

There is a need for data collection regarding pregestational maternal SARS-CoV-2 infection and its association with future pregnancies. Given the relative rarity of intraplacental transmission of SARS-CoV-2, we will need large epidemiological studies to answer these questions. We should also stay vigilant, monitoring past infections and the effects of emerging strains in assisted reproductive technologies (ART)^35^^,^^36^ and early pregnancy outcomes.

Although we need better data on miscarriages, it should be noted that there is no association between COVID-19 vaccination and miscarriages.^37^ It should be emphasised that we have robust evidence that vaccination against COVID-19 before or during early pregnancy is not associated with malformations, ruling out any role of COVID-19 vaccines in these increased rates of congenital abnormalities.^12^^,^^38^, ^39^, ^40^, ^41^, ^42^, ^43^ Of note, studies have demonstrated there were significant differences in eye, ear, face, and neck anomalies between the vaccinated and not vaccinated groups, showing vaccination protection from such anomalies.^44^ Similarly, it has been shown that COVID-19 vaccination is not related to non-syndromic orofacial cleft and might protect against having a child affected with such congenital anomalies.^45^ Thus, the data presented here should by no means be interpreted as associated with the vaccines, but the disease caused by SARS-CoV-2 infection.

Comprehensive systematic reviews and meta-analyses are essential to address these questions thoroughly, potentially offering valuable insights into mitigating neurodevelopmental risks and improving outcomes for children exposed to various prenatal factors. Developmental screening and follow-up of children with confirmed antenatal or neonatal SARS-CoV-2 exposure may also be relevant to identify those requiring early intervention.^46^

## Contributors

Viewpoint conceptualization, literature search, figures study design, and writing were led and coordinated by AS and AK. AS, VS, CMC and AK, contributed to the writing, visualisation, development and refinement of the manuscript. All authors endorsed the decision to submit it for publication.

## Declaration of interests

AK is Vice President of the RCOG. All other authors declare no competing interests.
